# Mining human periodic behaviors *via* tensor factorization and entropy

**DOI:** 10.7717/peerj-cs.1851

**Published:** 2024-01-31

**Authors:** Feng Yi, Lei Su, Huaiwen He, Tao Xiao

**Affiliations:** School of Computer Science, Zhongshan Institute, University of Electronic Science and Technology of China, Zhongshan, Guangdong Province, China

**Keywords:** Spatiotemporal data mining, Human periodic behaviors, Relative entropy, CP decomposition, Mobility intention

## Abstract

Understanding human periodic behaviors is crucial in many applications. Existing research has shown the existence of periodicity in human behaviors, but has achieved limited success in leveraging location periodicity and obtaining satisfactory accuracy for oscillations in human periodic behaviors. In this article, we propose the Mobility Intention and Relative Entropy (MIRE) model to address these challenges. We employ tensor decomposition to extract mobility intentions from spatiotemporal datasets, thereby revealing hidden structures in users’ historical records. Subsequently, we utilize subsequences associated with the same mobility intention to mine human periodic behaviors. Furthermore, we introduce a novel periodicity detection algorithm based on relative entropy. Our experimental results, conducted on real-world datasets, demonstrate the effectiveness of the MIRE model in accurately uncovering human periodic behaviors. Comparative analysis further reveals that the MIRE model significantly outperforms baseline periodicity detection algorithms.

## Introduction

Periodic behavior is a ubiquitous phenomenon in human society. On weekdays, individuals commute between workplaces and homes on workdays. On weekends, they follow weekly social or entertainment routines. Additionally, they engage in routine tasks such as individual income tax returns, birthday celebrations, or wedding anniversaries on a monthly or annual basis.

With the growing popularity of location-based services (LBSs) (Cho, Myers & Leskovec, 2011) and the rapid development of IoT devices, spatiotemporal datasets have become increasingly detailed, capturing a vast amount of human footprints. These datasets serve as valuable resources for analyzing human periodic behaviors, which have diverse applications in fields such as including crime prevention, intelligent transportation (Duan et al., 2022), epidemic prediction (Yu et al., 2023), privacy preservation (Xiong et al., 2014), and mass movement prediction (Shi et al., 2019a; Yuan et al., 2017b).

Essentially, mining human periodic behavior involves identifying activities that recur with specific, regular time intervals. In the past decade, several methods have been developed for mining human periodic behavior from spatiotemporal data (Cao, Mamoulis & Cheung, 2007; Li et al., 2010; Lian et al., 2015; Yuan et al., 2017b; Shi et al., 2019a; Lee & Jang, 2019; Chen et al., 2020; Zhang et al., 2020; Wang et al., 2022). However, most existing methods focus on the periodicity of locations, assuming that people regularly visit specific places. Nevertheless, driven by the personality trait of neophilia, individuals also show exhibit a propensity for novelty-seeking in human mobility, leading them to explore unvisited locations that are tailored to their interests (Lian et al., 2015). Consequently, some periodic behaviors, such as traveling and dining, can occur at various distinct locations. Therefore, human periodic behaviors may manifest at different and varying locations, which is common in many real-life scenarios.

In recent years, researchers have investigated mining human periodic behaviors based on location clusters with similar types (Yuan et al., 2017b; Ma et al., 2023) or semantics (Zhang, Lee & Lee, 2019). However, two significant barriers currently hinder the accurate mining of human periodic behaviors.

1. Locations associated with the same human periodic behavior can be widely dispersed, posing a challenge for existing mining methods.
2. Different periodic behaviors might occur at the same multi-functional area, leading to multiple periodic behaviors being observed at a single location.

**Figure 1:**
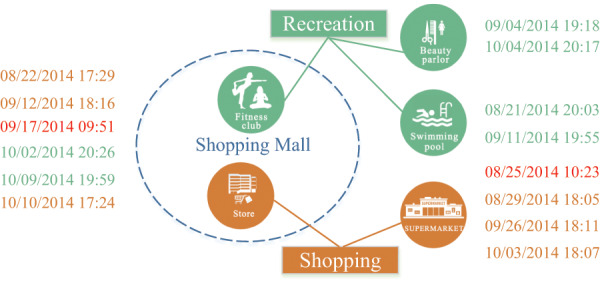
A history footprint of a user.

To illustrate these barriers, let’s consider a running example depicted in Fig. 1. Imagine a fitness club and a store located in the same shopping mall. Additionally, a supermarket, a beauty parlour and a swimming pool are scattered across various locations downtown. A user follows a routine of engaging in recreational activities like fitness, hairdressing, or swimming every Thursday night. After work on Fridays, he randomly visits either the store or the supermarket. In this scenario, two periodic behaviors: recreation and shopping, occur at the same location. However, distinguishing between them solely based on location data is challenging. Furthermore, the user might randomly choose between two markets that are significantly distant from each other. Consequently, mining these two human periodic behaviors based solely on location information is infeasible. This example highlights the limitations of existing periodic behavior mining methods in dealing with such complex situations.

To gain a comprehensive understanding of human mobility and behavior, it’s imperative to consider both spatial and temporal aspects when analyzing large-scale spatiotemporal datasets. A significant breakthrough in this domain is the concept of “mobility intentions”, sometimes referred to as “spatiotemporal topics” in literatures. These mobility intentions offer a way to identify patterns in spatiotemporal data that might not be immediately evident through simple spatial or temporal analysis. A mobility intention represents a specific combination of spatial and temporal elements associated with spatiotemporal data. Its primary goal is to uncover hidden structures within the data by capturing the intricate relationship between locations and times. Generally speaking, human mobility is fundamentally driven by specific mobility intentions, such as commuting, shopping, travels, and recreation. As the intrinsic factor for human mobility, these mobility intentions exhibit greater periodicity compared to locations (Yang et al., 2017).

Figure 1 illustrates periodic patterns associated with two mobility intentions: recreation and shopping, where the user exhibits periodic patterns, each with a weekly period. This observation suggests that the mobility intentions hold promising potential for modeling human periodic behaviors. However, mining periodic behaviors from spatiotemporal datasets based on these mobility intentions poses several challenges.

- **Inaccessibility of mobility intentions.** We lack direct access to mobility intentions, making the process of discovering these intentions from sparse individual records highly challenging.
- **User variability in the same location.** Different users meeting at the same location around the same time may have different mobility intentions. For instance, as illustrated in Fig. 1, within the same shopping mall, some individuals might be shopping, while others might be engaging in recreational activities. Designing an effective mapping from users’ footprints to their respective mobility intentions is a highly non-trivial task.
- **Detection of periodicity.** Detecting the periodicity of human periodic behaviors is challenging due to the uneven sampling and sparsity of spatiotemporal datasets. These datasets are often mixed with multiple periods and noise, making it difficult to discern the underlying patterns. Additionally, human periodic behaviors exhibit inherent complexity, with slight oscillations across different time intervals.

To address these challenges, we propose a novel model for mining human periodic behaviors, known as the “Mobility Intention and Relative Entropy (MIRE)”-based periodic behavior mining model, from the perspective of human mobility intentions. Our model offers an effective way to discover human periodic behaviors by utilizing these mobility intentions.

We first employ tensor decomposition techniques to extract human mobility intentions from a spatiotemporal dataset. Through comprehensive feature engineering, a multi-class classifier is trained to map individual footprints to specific mobility intentions. This mapping serves as the foundation for all subsequent steps in our model. With the mapping of footprints to mobility intentions established, we proceed to transform a user’s records into a sequence of mobility intentions. This sequence becomes the basis for our exploration of human periodic behaviors.

For the task of periodicity detection, we rely on a feasible approach of segmenting the sequence (Li, Wang & Han, 2012). Segmentation can effectively reveal patterns of periodicity. True periods tend to result in observations concentrated within specific time intervals, while an incorrect period leads to observations being scattered. To quantify this disorder, we turn to information theory (Haroutunian, 2011) and use entropy as a measure. However, direct comparing entropy between different potential periods can be problematic due to variations in macroscopic systems. To address this problem, we introduce relative entropy as our periodicity measurement. This approach allows us to effectively compare the disorder across different potential periods.

Once periodicity is established, an intriguing research challenge is how to evaluate human periodic behaviors. These behaviors can vary, with some oscillating slightly while others oscillate more substantially. To assess how closely a human periodic behavior aligns with the strict definition of periodicity in mathematics, we propose a coverage width-based criterion. This criterion provides a means for comparing and evaluating different human periodic behaviors.

The major contributions of this article are as follows:

1. We introduce an innovative model for mining human periodic behavior which is based mobility intentions derived through tensor factorization.
2. We propose a novel periodicity detection algorithm based on the relative entropy. Importantly, we provide a rigorous proof of the validity of this algorithm.
3. To the best of our knowledge, we are the first to propose a coverage width-based criterion that allows for the comparison of periodicity in human behavior.
4. Our contributions are supported by extensive experiments on both synthetic and real-world spatiotemporal datasets. The results demonstrate the effectiveness and precision of our proposed model compared to state-of-the-art models.

The remainder of this article is organized as follows. The “Preliminaries and Related Works” section covers related works and introduces a sequential representation of human periodic behavior with a mathematical formulation. The “Method” section details the core concepts of our proposed model. The “Experiment and Analysis” section presents detailed results from extensive experiments and provides in-depth analysis. Finally, we summarize our findings and conclude the article in the “Conclusions” section.

## Preliminaries and related works

This section formally defines the problem in the “Preliminaries” subsection and conducts a comprehensive review of prior research related to the mining of human periodic behaviors from spatiotemporal data in the “Related Works” subsection. Additionally, we categorize existing periodicity detection algorithms in the “Related Works” subsection to provide a more structured understanding of the field.

### Preliminaries

#### Problem statement: periodic behavior mining

Given a spatiotemporal dataset *D* consisting of *N* users. Let ${O_i}$ denote the collection of records for user ${u_i}$. Each record $o_i^j \in {O_i}$ is a two-tuple $o_i^j = (loc_i^j,t_i^j)$ indicating that ${u_i}$ visited location $loc_i^j$ at time $t_i^j$. The location $loc_i^j$ is a geographic coordinate. Let $o_i^k = (loc_i^k,t_i^k)$ and $o_i^{(k - 1)} = (loc_i^{(k - 1)},t_i^{(k - 1)})$ be the $k$-th and the $(k - 1)$-th record of user ${u_i}$, respectively. Let $o_i^{{k^\prime }} = (loc_i^k,t_i^{{k^\prime }})$ be the previous record for user ${u_i}$ to appear at the same location $loc_i^k$ as in ${O_i}$.

As previously mentioned, we aim to mine human periodic behaviors based on mobility intentions rather than locations. In this context, a mobility intention, denoted as $m$, represents a specific combination of spatial and temporal elements associated with spatiotemporal data. Essentially, a mobility intention, represented by ${m_s}$, acts as a probability distribution that helps explain the reasons behind a user’s presence at a specific location $loc$ at a specific time $t$. Let ${\cal M} = \{ {m_s}|1 \le s \le M\}$ denote the set of *M* mobility intentions.

We use a binary sequence $X = \{ I(t)|t = 0, \cdots ,n - 1\}$ to denote a time sequence, where $I(t) = 1$ if and only if user ${u_i}$ exhibits the mobility intention $m_i^s$ at timestamp $t$, otherwise $I(t) = 0$.

In general, a mobility intention $m_i^s$ for user ${u_i}$ is considered periodic with a period ${T_0}$ if ${u_i}$ exhibits $m_i^s$ in every ${T_0}$ time units. However, in real-world scenarios, human periodic behaviors may not unfold with the same precisely the same period during different cycles; they may exhibit oscillations across various intervals. For instance, as shown in Fig. 1, the time at which a user engages in shopping every Friday evening fluctuates between 17:40 to 18:20. Consequently, we can formally define human periodic behavior as follows.

**Definition 1 Human Periodic Behavior**: Suppose ${T_0} > 1$ and $0 \le {t_0} \le {T_0}$, for any $0 \le {t^ \star }{\mathrm{ \lt}}{T_0}$

(1) $$I({t^ \star }) = \left\{ {\matrix{ {1,\qquad {t^ \star } = {t_0}} \hfill \cr  {0,\qquad {\mathrm{otherwise}}} \hfill \cr  } } \right..$$

If there is one and only one timestamp ${t^\prime } \in [{t_0} - \delta + k{T_0},{t_0} + \delta + k{T_0}]$ of *X* which satisfies ${\mathrm{I}}({t^\prime }) = {\mathrm{I}}({t_0})$ for $k = 0,1, \ldots ,\quad \,(n - 1,{T_0})$, the binary sequence *X* is a *periodic behavior binary sequence* with the period ${T_0}$.

Here ${t_0}$ is the average timestamp that $m_i^s$ happened in a true period ${T_0}$. $\delta$ is a relative buffer that enables $m_i^s$ to oscillate in the interval $[{t_0} - \delta + k{T_0},{t_0} + \delta + k{T_0}]$ instead of being fixed at an exactly timestamp ${t_0} + k{T_0}$. If $\delta = 0$, the human periodic behavior is called a *strictly periodic behavior*.

Given a spatiotemporal dataset *D*, the aim of periodic behavior mining includes:

1. Extracting a set of mobility intentions ${M}$;
2. Training a classifier for mapping footprints to mobility intentions;
3. Identifying the true period $T_0^{(i,s)}$ associated with a mobility intention ${m_s}$ for user ${u_i}$.

### Related works

#### Period detection algorithm

Period detection has been a long-standing research issue in time series data mining. Common and widely used methods for period detection include Fast Fourier transform (FFT) and Autocorrelation. However, they do not perform well on noisy observations (Li et al., 2010). Several researchers have proposed alternative period detection algorithms, such as WARP (Elfeky, Aref & Elmagarmid, 2005), Lomb-Scargle periodogram (Glynn, Chen & Mushegian, 2006), the combination of the autocorrelation function and FFT (Berberidis et al., 2002; Li et al., 2010).

As LBSs continue to gain widespread adoption, a vast amount of human footprints have been meticulously documented in spatiotemporal datasets, which essentially represent time series data. Some researchers have identified periodicity in user check-in locations within these spatiotemporal datasets (Cao, Mamoulis & Cheung, 2007; Li et al., 2010). Although the algorithms mentioned above have made certain progress in period detection in time series data mining, their performance is often affected when applied to spatiotemporal datasets due to data sparsity and incompleteness. To address these challenges, numerous novel algorithms for periodicity detection have been proposed.

For instance, Yuan et al. (2017b) modeled the time gap between two consecutive records as a univariate Gaussian distribution and employed a probability generative model for periodicity detection. Ghosh, Lucas & Sarkar (2017) proposed a particle filter algorithm to detect periodicity in the presence of noise. He et al. (2018) utilized automated machine learning for periodicity extraction. Galbrun et al. (2018) employed the minimum description length criterion is used to evaluate candidate periodicity. Song et al. (2022) introduced an improved version of Dynamic Time Warping (DTW) for more precise periodicity detection.

Furthermore, to address the challenges of uncertain and oscillating periodic human behavior, a partition algorithm for periodicity detection was introduced (Li, Wang & Han, 2012) and later enhanced by Yuan et al. (2017a). Li, Wang & Han (2012) proposed a probability measurement to evaluate the likelihood of various potential period *T*, selecting the period with the highest likelihood as the true period. Building upon this work, our algorithm determines the true period using relative entropy instead of likelihood. This novel algorithm mitigates issues related to favoring short periods and enhances robustness.

#### Mobility intentions

In theory, researchers have proven that $93\%$ of human mobility is regular and predictable (Song et al., 2010). However, identifying explicit periodicity in individuals’ check-in locations within spatiotemporal data often poses challenges. To address this issue, researchers have turned their attention to investigating the periodicity of more abstract aspects of human mobility. These include periodicity through location clustering (Yuan et al., 2017b; Ma et al., 2023) and location semantic periodicity (Zhang, Lee & Lee, 2019). Among these abstract aspects, the concept of mobility intention has been introduced to explain why individuals appear at specific locations at particular times (Hu, Jamali & Ester, 2013; Yuan et al., 2013; Liu et al., 2015). This concept is also occasionally denoted as spatiotemporal topics in the literatures. Mobility intention encompasses a combination of factors such as time and place, resulting in more pronounced periodicity compared to the solo consideration of location or location semantics (Zhao, Koutsopoulos & Zhao, 2020).

Several algorithms have been developed for extracting mobility intentions. While some are rooted in traditional Latent Dirichlet Allocation (LDA) techniques (Hu, Jamali & Ester, 2013; Yang et al., 2017; Chen et al., 2019; Zhao, Koutsopoulos & Zhao, 2020), the increasing adoption of tensor factorization-based models is noteworthy. Tensors can be considered as a generalization of matrices and tensor factorization can be regard as higher-order extensions of the matrix singular value decomposition (SVD) Tensor factorization is able to extract latent features in dataset, as well as reduce data dimensionality. The tensor-based method has the capability to model multifaceted data and effectively handle correlations among multiple dimensions. As a result, it has been successfully applied to the analysis of heterogeneous spatiotemporal data, especially for extracting mobility intentions.

For example, Yan et al. (2018) considered both the spatiotemporal aspects and user information to mine bicycle usage patterns among different age groups from bike-sharing data. Additionally, Shi et al. (2019b) leveraged multi-source mobility datasets and Point of Interest (POI) data to construct two region-feature-time tensors for mobility intentions extraction, unveiling insights into people’s travel intentions and region functionalities. Liu et al. (2021) propose an augment nonnegative tensor factorization-based model that combine mobility semantics and inherent location information for mobility intention identifying. TPFlow (Liu, Xu & Ren, 2019) introduced a pioneering piecewise rank-one tensor decomposition, facilitating automated partitioning and the extraction of multi-dimensional mobility intentions from spatiotemporal data. These tensor-based methods have significantly advanced the extraction of mobility intentions.

Tensor factorization typically involves methods such as tensor train, Tucker factorization and Candecomp/Parafac (CP) decomposition, among others (Rehman et al., 2016). Tensor train is a simple and robust method for model compression (Oseledets, 2011) and has been studied extensively for deep learning application domains, such as computer vision (Yang, Krompass & Tresp, 2017) and natural language processing (Hrinchuk et al., 2020). Tucker factorization decomposes a tensor into a set of factor matrices and a smaller core tensor. As a rule of thumb, it is usually advised to use Tucker factorization for subspace estimation, compression, and dimensionality reduction. It is commonly employed in research fields such as signal processing (Hu, Deng & Yuan, 2020) and image processing (Hatvani et al., 2021).

The CP decomposition (Kolda & Bader, 2009) stands as one of the most widely adopted tensor analysis methods. It effectively decomposes a tensor into a summation of rank-one components, allowing it to reveal latent structures within spatiotemporal datasets. Researchers have harnessed this method to analyze various types of spatiotemporal data, such as location-based social network (LBSN) data (Luan et al., 2017; Shi et al., 2019b), bikesharing data (Yan et al., 2018) traffic flow data (Han & Moutarde, 2016; Liu et al., 2021; Liu, Xu & Ren, 2019). For instance, Takeuchi, Kawahara & Iwata (2017) modeled traffic flow data as a three-dimensional tensor, where each dimension corresponded to days, hours, and geographical locations, respectively. They proceeded to decompose this tensor into three non-negative rank- $1$ factors, essentially feature descriptors, to elucidate the daily, hourly, and spatial trend and distribution patterns. The CP decomposition is also suitable for extracting mobility intentions in this article.

## Method

### Mobility intentions extraction

As mentioned earlier, mobility intentions are typically latent but exhibit a strong degree of spatiotemporal regularity. For example, the act of commuting, a fundamental mobility intention found in many spatiotemporal datasets, explains why an individual consistently arrives at the workplace around $9$ am on workdays. Before delving into the analysis of mobility intentions, it is essential to identify the specific mobility intentions that are inherent in a given spatiotemporal dataset.

In this article, we employ the CP decomposition to extract mobility intentions from a given spatiotemporal dataset. To apply the CP decomposition, we construct a three-dimensional tensor ${Y}$ comprising location, hour, and day. This tensor forms the basis for our analysis. The element ${y_{{r_i},{t_j},{d_k}}}$ of the three-way tensor ${ Y} \in {R^{M \times H \times D}}$ can be computed as

(2) $${y_{{r_i},{t_j},{d_k}}} = {{Count({r_i},{t_j},{d_k})} \over {\sum\nolimits_{q = 1}^L C ount({r_q},{t_j},{d_k})}}$$

where ${r_i}$, ${t_j}$, and ${d_k}$ are the index of the location, the time bin and the day of month, respectively; *L* is the total number of locations, and $Count({r_i},{t_j},{d_k})$ is the number of users who appeared at location ${r_i}$ at time ${t_j}$ on the ${d_k}$-th day.

In the CP decomposition, the tensor ${Y}$ is factorized into a sum of rank one component tensors ${{\bf{Y}}_g}$:

(3) $${Y} \approx \sum\nolimits_{g = 1}^G {{\lambda _g}} {{\bf{Y}}_g}.$$

The rank one tensor ${{\bf{Y}}_g}$ is outer product of three vectors:

(4) $${{\bf{Y}}_g} = {{\bf{r}}_g}\circ {{\bf{t}}_g}\circ {{\bf{d}}_g}$$

The components of ${{\bf{r}}_g}$, ${{\bf{t}}_g}$ and ${{\bf{d}}_g}$ represent the probability distribution of ${{\bf{Y}}_g}$ on locations, time bins and days of month, respectively. ${{\bf{Y}}_g}$ can be used to explain why a user appeared in one location at a special timestamp. Hence, the ${{\bf{Y}}_g}$ is identified as one mobility intention ${m_s}$ in this article.

Because CP decomposition can effectively decomposes a tensor into a summation of rank-one tensors, each of these rank-one tensors can be treated as a mobility intention. Therefore, CP decomposition is chosen for extracting mobility intentions from spatiotemporal datasets in this article.

**Figure 2:**
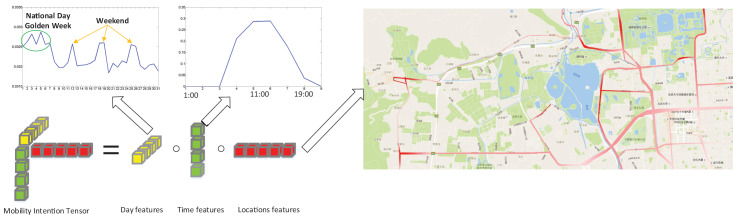
A example of a rank-one tensor.

In Fig. 2, we present an example of a rank-one tensor, denoted as ${\bf{Y}}_g^ \star$, extracted from the Beijing Bus Smart Card (BBSC) dataset, which will be used in our later experiment. The figure on the right highlights bus stops with higher values in a deeper shade of red, primarily concentrated in the northwest area of Beijing. These bus stops highlighted in deeper red are the locations where the mobility intention ${\bf{Y}}_g^ \star$ is more likely to appear. Notably, these bus stops are situated close to popular recreation areas such as the Summer Palace, the Yuanmingyuan Imperial Garden and the Fragrant Hills park. Analyzing the day features reveals that this mobility intention exhibits weekly periodicity. Additionally, during the Chinese National Day Golden Week (October $1$ st to $7$ th), we observe a surge in activity at the bus stops highlighted in deeper red. Focusing on the time features reveals a clear peak at around $11:00$, coinciding with the time when most people arrive at the bus stops with a higher probability. Based on this analysis, we can categorize this rank-one tensor shown in Fig. 2 as a mobility intention related to “recreation”.

To reveal the set of mobility intentions hidden within a dataset *D*, we employ the CP decomposition. To detect the period of a particular mobility intention, we require a function that maps records to those mobility intentions. In this context, each mobility intention is treated as a distinct class. Consequently, every record ${f_i}$ corresponds to one specific mobility intention ${m_s} \in {M}$, and can be thought of as belonging to a particular class. Thus, the process of mapping records to mobility intentions is transformed into a multi-class classification problem. To ensure high performance in multi-class classification, we perform comprehensive feature engineering and model training. After analyzing the vectors of the rank-one tensor and performing feature engineering, we introduce three distinct types of features: spatial features, hourly features, and daily features.

**Spatial Features**

1. *Location entropy*. Location entropy measures the popularity of a location. For a location $lo{c_k}$, its location entropy is defined as:
(5) $$ H(loc_k^i) = - \sum\limits_i^{} {{p_i}} \ln {p_i}$$
  where ${p_i}$ is the proportion that the number of times for user ${u_i}$ to visit location $lo{c_k}$ among all users who visited the same location $lo{c_k}$.
2. *Location type*. Location type indicates the category of location $lo{c_k}$, such as bar, mall and park. Location type can be obtained from LBS’s application program interface (API), such as Google Places API (https://developers.google.com/places/) or Sina Weibo API (http://open.weibo.com/).
3. *Distance*. Here, the distance refers to the Euclidean linear distance between location $loc_k^i$ and its last location $loc_{k - 1}^i$ visited by the same user ${u_i}$.

**Hour Features**

1. *Hour*. The hour is the hour of time $t_k^i$ in a day which is denoted by $\{ 0,...,23\}$.
2. *Stay time*. The stay time is time interval between two continuous footprints of an user, *i.e*., $t_k^i - t_{k - 1}^i$.
3. *Time span*. The time span is time interval between two footprints which occurred at the same location $loc_k^i$, *i.e*., $t_k^i - t_{{k^\prime }}^i$.

**Day Features**

1. *Day of week*. The day of week refers weekday of $t_k^i$. It is denoted by $\{ 0,...,6\}$ which means Sunday to Saturday.
2. *Day of month*. The day of month is day in month of $t_k^i$. It is denoted by $\{ 1, \cdots ,31\}$.
3. *Day Type*. The day type refers to the category of $t_k^i$. There are three categories in this article, workday, short break holidays and long holidays.

In conclusion, utilizing these three types of features, we utilize the Adaboost classifier from the sklearn (https://scikit-learn.org/) (Pedregosa et al., 2011) to train an Adaboost model, which is responsible for mapping each record into a specific mobility intention.

**Figure 3:**
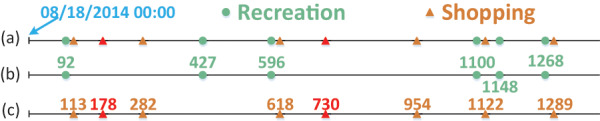
The mixed sequence and spitted mobility intention sequence corresponding to Fig. 1.

Through the application of the Adaboost model, the records in our running example from Fig. 1 can be classified into a mixed sequence representing two distinct mobility intention: “recreation” and “shopping”, as depicted in Fig. 3A. Subsequently, as illustrated in Figs. 3B and 3C, we acquire separate time sequences for each mobility intention. The implementation of the Adaboost classification model enables the effective separation of multiple mixed periods.

### Period identification

**Figure 4:**
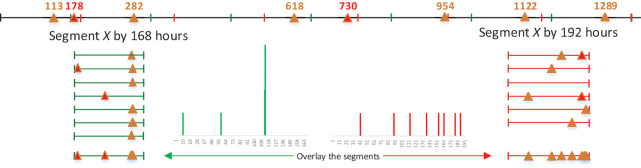
Illustration example of folded time sequence *X* in Fig. 3C.

To detect periodicity within noisy, unevenly sampling and incomplete observations, a feasible way involves dividing the sequence *X* into segments, as proposed by Li, Wang & Han (2012). As depicted in Fig. 4, It’s evident that the majority of observations fall into a specific time interval when the timeline of a given mobility intention is segmented based on the true period.

In general, if we consider a binary sequence *X* with a length $n$ segmented by a trial period *T*, we can determine the mobility intention count occurring within each timeslot in *T*.

(6) $${S_i}(T) = \{ t|\quad  \,(t,T) = i \wedge {\mathrm{I}}(t) = 1\} ,\;t = 0,1, \ldots ,n - 1,\;i = 0,1, \ldots ,T - 1$$

The probability at each timeslot in *T* is then

(7) $${p_i}(T) = {{|{S_i}|} \over {\sum\limits_{j = 0}^{T - 1} | {S_j}|}},\;i = 0,1, \ldots ,T - 1$$

Apparently, ${p_i}(T)$ follows $\sum\nolimits_{i = 0}^{T - 1} {{p_i}} (T) = 1$.

An intuitive method for detecting periodicity involves calculating ${p_i}(T)$ for each candidate *T* and select the candidate with the highest probability as a criterion for identifying the period.

(8) $${\gamma _X}({T_0}) = \max {p_i}(T).$$

The true period under consideration, denoted as ${T_0}$, corresponds to a candidate period *T* which yields the maximum value of ${\gamma _X}(T)$, as illustrated in Fig. 4.

**Figure 5:**
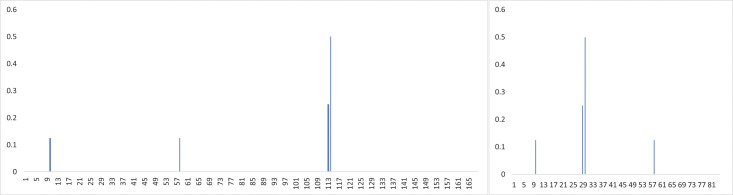
The measurement of ${\gamma _X}(T)$ favours shorter periodicity.

However the measurement of ${\gamma _X}({T_0})$ has a drawback of favouring shorter periodicity. In general, when more observations fall into the same timestamps in a candidate *T*, it results in a lower value of ${\gamma _X}(T)$. Given that the timeline is folded in half, two scenarios arise: either more observations coincide at the same timestamps, resulting in a higher value of ${\gamma _X}(T)$, or the observations’ distribution across timeslots remains unchanged, leading to the same value of ${\gamma _X}(T)$. As shown in Fig. 5, after folding the timeline in half, the value of ${\gamma _X}(T/2)$ is equal to ${\gamma _X}(T)$.

Furthermore, let’s define $r$ as the ratio of the time interval in which most observations fall after segmentation by the true period. The above method’s performance drops drastically and is not robust when the ratio $r$ is smaller than $0.3$, which is just in line with many real-life scenarios. For example, consider the situation where the true period ${T_0} = 24$ and $r = 0.125$. In this case, all observations will fall in an interval with length of $3$ after being segmented by the true period ${T_0}$, and the corresponding accuracy of the method described above is no more than $0.3$. However, in most human mobile behavior scenarios, the value of $r$ is much smaller than $0.125$. Therefore, a new measurement is required to address these limitations.

Suppose we have a binary sequence of behavior *X* with a true period ${T_0}$. According to **Definition 1**, the observations in this sequence should ideally fall within a compact interval of $[{t_0} - \delta ,{t_0} + \delta ]$ when *X* is segmented by its true period ${T_0}$. However, when *X* is incorrectly by another candidate period ${T_f}$, these observations might be dispersed across scattered intervals. For instance, as shown in Fig. 4, when we segment the binary sequence *X* by its true period of $168$ h (a week), the observations are concentrated around $114$. In contrast, if we segment *X* by $192$ h, the observations are disordered and spread across a wide time interval. Observations are notably more ordered when *X* is segmented by the true period ${T_0}$, in contrast to the disordered pattern that emerges when *X* is segmented by an incorrect period.

To quantitatively assess this disorder, we turn to the concept of entropy, which is commonly used in physics to measure the chaos or randomness of a system. Consider that different candidate periods can be likened to different systems; we can employ entropy as a metric to quantify the level of disorder across these candidate periods. The entropy of a candidate period is defined as follows:

(9) $$H(T) = - \sum\limits_{i = 0}^{T - 1} {{p_i}} (T)\log {p_i}(T).$$

In our context, a lower entropy value $H(T)$ signifies that observations are more concentrated when segmented by the candidate period *T*, while a higher entropy value suggests more dispersion. Therefore, entropy can be served as a useful metric for periodicity detection. Notably, the entropy is at its minimum when *X* is segmented by its true period ${T_0}$, reflecting a highly ordered and periodic pattern.

However, the entropy metric also exhibits a preference for shorter periodicity, which poses a challenge similar to the intuitive method. For instance, consider a periodic behavior binary sequence *X* generated from Definition 1 with an candidate period ${T_0}$ which is even. If ${T_0}/2$ is not included in the compact interval $[{t_0} - \delta ,{t_0} + \delta ]$, then according to Eq. (9), we have $H({T_0}) = H({T_0}/2)$. Consequently, one might mistakenly conclude that ${T_0}/2$ is the true period.

This issue arises because the entropy assesses the degree of disorder of a potential period independently. However, our objective is to compare the disorder of various potential periods, and the entropy of different potential periods should not be directly compared. As previously mentioned, a more concentrated distribution of observations segmented by a candidate periodicity *T* indicates that *T* is closer to the true period. Conversely, when the distribution of observations approaches uniformity, *T* is an incorrect period. In an extreme case, a mobility intention might occur at nearly every timestamp with almost equal probability when *X* is segmented by an incorrect period ${T_f}$. In such an extreme scenario, no true period exists which means *X* has no periodicity at all. Therefore, we can employ the discrepancy in entropy between ${p_i}(T)$ and the uniform distribution on a potential period *T* as the periodic measurement. The discrepancy of a candidate period *T* is defined as follows:

$\eqalign{ H({U_T}) - H(T) {=  - \sum\limits_{i = 0}^{T - 1} {{1 \over T}} \log {1 \over T} + \sum\limits_{i = 0}^{T - 1} {{p_i}} (T)\log {p_i}(T)\\ = \log T\sum\limits_{i = 0}^{T - 1} {{p_i}} (T) + \sum\limits_{i = 0}^{T - 1} {{p_i}} (T)\log {p_i}(T)\\ = \sum\limits_{i = 0}^{T - 1} {{p_i}} (T)\log {{{p_i}(T)} \over {1/T}}}}$

The last formula provides the definition of relative entropy between ${p_i}(T)$ and the uniform distribution on *T* which is also known as the Kullback-Leibler divergence. Therefore, the above formula can also be written in the following form:

(10) $${\mathrm{KL}}(T) = H({U_T}) - H(T) = \sum\limits_{i = 0}^{T - 1} {{p_i}} (T)\log {{{p_i}(T)} \over {1/T}} = \log T - H(T)$$

Therefore, when dealing with a periodic behavior binary sequence *X* with unknown period, if the probability distribution ${p_i}(T)$ exhibits a sharper peaked and the discrepancy from the uniform distribution on *T* is more pronounced, *T* is closer to the true period ${T_0}$. This leads us to the following lemma, which asserts that the relative entropy reaches its maximum at the true period ${T_0}$.

**Lemma 1** : If a binary sequence *X* is generated periodically according to a categorical distribution ${\mu }_0^\prime$ with a period ${T_0}$, then for any $T \ge 2,T \in \mathbb{N}$, we have

(11) $$\mathop {\lim }\limits_{n \to \infty } {\mathrm{KL}}({T_0}) \ge \mathop {\lim }\limits_{n \to \infty } {\mathrm{KL}}(T)$$

The following is proof of Lemma 1. Based on Definition 1, we assume that a periodic time sequence *X* is generated from a categorical distribution for some period ${T_0}$, and the parameter of this distribution is ${\mu}{_0} = ({\mu _0},{\mu _1}, \ldots ,{\mu _{{T_0} - 1}})$, where $\sum\nolimits_{i = 0}^{{T_0} - 1} {{\mu _i}} = 1$. Let ${I_v} = [{t_0} - \delta ,{t_0} + \delta ] \subseteq [1,{T_0}]$. From the Definition 1, it’s clear that the probability of ${\mu}{_0}$ falling in ${I_v}$ are greater than those falling in $[1,{T_0}]/{I_v}$. That is to say that ${\mu _g} \gg {\mu _h},g \in {I_v},h \in [1,{T_0}]/{I_v}$,

We use ${T_0}$ and *T* to denote the true period and one candidate period. For an interval $[0,T \times {T_0} - 1]$, it’s obvious that this interval contains *T* periods of ${T_0}$ or ${T_0}$ periods of *T*. Let ${p_{i,j}}$ be the $i$-th position of period *T* in the $j$-th segment. Then we have:

(12) $${p_{i,j}} = {\mu _{(i + j \cdot T)\;\;\;\, \,\text {mod}\,{T_0}}}$$

The $i$-th position’s parameter of period *T* is:

(13) $$\mu _i^\prime (T) = {1 \over T}\sum\limits_{j = 0}^{{T_0} - 1} {{p_{i,j}}}$$

The relative entropy of period *T* is

(14) $${\mathrm{KL}}(T) = \ln T + \sum\limits_{i = 0}^{T - 1} {{p_i}} (T)\ln {p_i}(T) = - {1 \over T}\sum\limits_{i = 0}^{T - 1} {\sum\limits_{j = 0}^{{T_0} - 1} {{p_{i,j}}} } \left( {\ln \sum\limits_{j = 0}^{{T_0} - 1} {{p_{i,j}}} } \right)$$

For ${\mu _g} \gg {\mu _h},g \in {I_v},h \in [0,{T_0} - 1] - {I_v}$, the relative entropy of period ${T_0}$ is

(15) $${\mathrm{KL}}({T_0}) = \sum\limits_{j = 0}^{{T_0} - 1} {{\mu _j}} \ln {\mu _j} + \ln {T_0} \ge \sum\limits_{k \in {I_v}}^{} {{\mu _k}} \ln {\mu _k} + \ln {T_0}$$

Then,

$ {\mathrm{KL}}({T_0}) - {\mathrm{KL}}(T) \ge \ln {T_0} + \sum\limits_{k \in {I_v}}^{} {{\mu _k}} \ln {\mu _k} - {1 \over T}\sum\limits_{i = 0}^{T - 1} {\sum\limits_{j = 0}^{{T_0} - 1} {{p_{i,j}}} } \left( {\ln \sum\limits_{j = 0}^{{T_0} - 1} {{p_{i,j}}} } \right)\\ = \ln {T_0} + {1 \over T}\sum\limits_{i = 0}^{T - 1} {\sum\nolimits_{j = 0}^{{T_0} - 1} {\left\{ {{1 \over {{T_0}}}\sum\nolimits_{k \in {I_v}}^{} {{\mu _k}} \ln {\mu _k} - {p_{i,j}}\left( {\ln \sum\nolimits_{j = 0}^{{T_0} - 1} {{p_{i,j}}} } \right)} \right\}} } \\ \ge \ln {T_0} + {1 \over T}\sum\limits_{i = 0}^{T - 1} {\sum\limits_{j = 0}^{{T_0} - 1} {{p_{i,j}}} } \ln {{{\mu _{{\mathrm{min}}}}} \over {{1 \over T}\sum\nolimits_{j = 0}^{{T_0} - 1} {{p_{i,j}}} }}\\  \ge \ln {T_0} + {1 \over T}\sum\nolimits_{i = 0}^{T - 1} {\sum\limits_{j = 0}^{{T_0} - 1} {{p_{i,j}}} } \ln {1 \over {{T_0}}} = \ln {T_0} + \ln {1 \over {{T_0}}} = 0          $

To mine ${T_0}$, compute ${\mathrm{KL}}(T)$ and select the value of *T* that maximizes ${\mathrm{KL}}(T)$ as the true period. The pseudo code of periodicity detection is shown in Algorithm 1.

**Algorithm 1:** Relative entropy based periodicity detection algorithm.

**Input:** a binary mobility intention sequence *X*, Maximal potential period *T*_*max*_
**Output:** the true period *T*_0_ of *X*
1: ${T_0} = 2$, $RH = 0,T = 2$
2: **while** $T \le {T_{max}}$ **do**
3: compute ${S_i}(T)$ as Eq. (6)
4: compute ${p_i}(T)$ as Eq. (7)
5: compute ${\mathrm{KL}}(T)$ as Eq. (10)
6: **if** $RH{\mathrm{< KL}}(T)$ **then**
7: $RH = {\mathrm{KL}}(T)$
8: ${T_0} = T$
9: **end if**
10: **end while**
11: **return** *T*_0_

The mining of a user’s periodic behaviors allows us to predict the user’s future locations. Specifically, for a given user $u_i$, his history records, and a target time $t_f$, we can identify which periodic behavior $t_f$ belongs to. Subsequently, we use the most frequent location within the target periodic behavior as the prediction result.

### The quantitative assessment of periodicity

Human behavior periodicity plays a crucial role in predictive applications, such as forecasting future user activities or movements. The accuracy of these predictions is inherently tied to the quality of the underlying human behavior periodicity. An ideal “good” periodic human behavior, characterized by its strict periodicity, leads to superior predictive performance. Predictions generated from such behavior are precise, resembling specific points on the timeline with high confidence levels. In contrast, a “bad” periodic human behavior exhibits inferior predictive performance, resulting in broader prediction intervals with lower confidence. But how do we measure what makes a periodic human behavior “good” or “bad”?

To assess prediction performance based on human behavior periodicity, we must define quantitative measure for assessment of periodicity. As aforementioned, in a periodic binary sequence, observations are expected to cluster within a narrow interval when partitioned with the true period. In an ideal strictly periodic behavior, which represents the highest quality binary sequence, all observations fall in a interval characterized by a single timestamp. It’s evident that a narrower interval with more observations aligning within it indicates a “good” periodic human behavior. On the contrary, if the interval is wide and contains fewer observations, the periodic behavior is considered “less favorable”. Therefore, a “good” periodic human behavior is one in which the interval is narrow and contains a substantial number of observations.

To quantify and evaluate this distinction, we employ the Coverage Width-Based Criterion (CWC) (Khosravi et al., 2011), a method that doesn’t rely on specific references. The CWC assesses periodic behavior by considering a target interval ${I_c}$ containing observed data points, the count of observations within this interval ${C_f}$, and the total number of observations in the sequence ${C_o}$. The interval coverage probability (ICP) is defined as follows:

(16) $$ICP = {{{C_f}} \over {{C_o}}}$$

The definition of CWC is

(17) $$CWC = NIW \cdot \left( {1 + {g^ \star }{e^{ - {\eta ^ \star }(ICP - {\mu ^ \star })}}} \right)$$

The NIW is normalized interval width which refers to the ratio of ${C_f}$’s width to true periodicity width, *i.e*.,

(18) $$NIW = {{{C_f}} \over {{T_0}}}$$

The constants ${\eta ^ \star }$ and ${\mu ^ \star }$ in 17 are two hyper parameters. ${\mu ^ \star }$ is a threshold related with ICP. Some observations should not fall into the target interval ${I_c}$ after partition for noises or outliers. Therefore, the value of ICP will be less than $1$ in reality. We assumed that all observations from human periodic behavior fall into the target interval ${I_c}$ when the value of the ICP is greater than the threshold ${\mu ^ \star }$. ${\eta ^ \star }$ is a coefficient determining how much penalty is assigned to ICP with a low value. It magnify the difference between ICP and ${\mu ^ \star }$. In this article, ${\eta ^ \star }$ is set to $2$ and ${\mu ^ \star }$ is set to $0.95$. ${g^ \star }$ is a function related with ICP and is given by the following step function:

(19) $${g^ \star } = \left\{ {\matrix{ {0,} & {ICP \ge {\mu ^ \star }} \cr  {1,} & {ICP\lt{\mu ^ \star }.} \cr  } } \right.$$

This function indicates that CWC will be affected by ICP only when the value of ICP is less than the threshold ${\mu ^ \star }$.

There are two parts in Eq. (17). The first part is NIW which is related with width of target interval ${I_c}$, and the other part is ICP which is affected by the number of observations that fall into the interval. If ICP is greater than ${\mu ^ \star }$ which means “all observations” fall in the target interval ${I_c}$, CWC is only decided by the width of ${I_c}$, *i.e*., NIW. The narrower the ${I_c}$ is, the smaller the CWC’s value is. On the other side, the value of CWC is much greater if ICP’s value is far less than ${\mu ^ \star }$. The criteria for a “good” human periodic behavior is narrow target interval with more observations fallen in. Therefore, the smaller the value of CWC, the “better” the human periodic behavior line, that means the human periodic behavior has the more obvious periodicity.

However, for some specific human periodic behavior, these two parts in Eq. (17) can be conflicting. When choosing a narrow interval, ICP is often small. Calculating CWC for a human periodic behavior requires the careful determination of IPC and NIW. To address this issue,we introduce a new CWC computing algorithm outlined in Algorithm 2.

**Algorithm 2:** The CWC computing algorithm.

**Input:** a binary mobility intention sequence *X*, the true periodicity *T*_0_
**Output:** the CWC’s value of the binary mobility intention sequence *X*.
1: $CW{C_{out}} = {T_0}$, ${C_f} = 1$
2: compute ${S_i}({T_0})$ as Eq. (6)
3: Sort ${S_i}({T_0})$ by descending
4: **while** ${S_i}({T_0}) > 0$ **do**
5: compute *icp* as Eq. (16)
6: compute *niw* as Eq. (18)
7: compute *cwc* as Eq. (17)
8: **if** $cwc< CW{C_{out}}$ **then**
9: $CW{C_{out}} = cwc$
10: **end if**
11: **end while**
12: **return** *CWC*_*out*_

For a strictly periodic behavior, all observation fall in one time stamp. Hence, the value of strictly periodic behavior’s $I_c$ is 1, its *ICP* is 1 and its CWC is

$CW{C_{sp}} = {1 \over {{T_0}}} \cdot \left( {1 + 0 \times {e^{ - 2 \times (1 - 0.95)}}} \right) = {1 \over {{T_0}}}$

In the running example presented in this article, the periodicity for the recreation and shopping sequence is $24$ and $168$ respectively, with corresponding CWC values as follows:

$ CW{C_{recreation}} = {2 \over {24}} \cdot \left( {1 + 0 \times {e^{ - 2 \times (1 - 0.95)}}} \right) = 0.083 \\  CW{C_{shopping}} = {1 \over {168}} \cdot \left( {1 + 1 \times {e^{ - 2 \times (0.75 - 0.95)}}} \right) = 0.018  $

The result shows that the periodicity of shopping is better than recreation.

## Experiment and analysis

In this section, we will evaluate the performance of the proposed periodicity detection algorithm on synthetic datasets with varying parameters. Additionally, a case study will be presented to illustrate its effectiveness in mining human periodic behaviors.

### Periodicity detection on synthetic time series data

#### Experimental settings

**Data Generation: **In order to evaluate the performance of the proposed periodicity detection method, we generate a set of synthetic datasets using the following steps as suggested in Li, Wang & Han (2012):

1. Given a fixed period ${T_0}$, for example, ${T_0} = 24$. A periodic segment ${X_{frac}}$ is a Boolean sequence of length ${T_0}$, with one value is $1$ indicating having the mobility intention, and all other values are set to $0$. The time ${t_i}$ around ${t_0}$ when a mobility intention occurred is modelled by the Gaussian distribution.
(20) $$p({t_i}) = {1 \over {{{(2\pi {\sigma ^2})}^{1/2}}}}\exp \left\{ { - {1 \over {2{\sigma ^2}}}{{({t_i} - {t_0})}^2}} \right\},$$
  where the ${\sigma ^2}$ is the variance.
2. The periodic segment ${X_{frac}}$ repeats *N* times to generate the complete sequence ${X_{comp}}$.
3. Sample the complete sequence with the sampling rate $\delta$. It means that the value $1$ in each period segment can be observed with probability $\delta$. Then we get the sequence ${X_{samp}}$ with sampling from ${X_{comp}}$.
4. We randomly flip one value of $0$ to $1$ in each period segment with noise rate $\gamma$ from ${X_{samp}}$ and obtain the synthetic dataset ${X_{test}}$.

In the experiment, the default values of above parameters are: ${T_0} = 24$, ${t_0} = 12$, $\sigma = 1$, $N = 120$, $\delta = 0.2$ and $\gamma = 0.1$.

**Methods for Comparison:** Three baseline models are chosen for performance comparison: FFT, ePeriodicity (Li, Wang & Han, 2012) and Periodic Region Detection(PRED) (Yuan et al., 2017b).

**Fast Fourier Transform (FFT)** The result period corresponds to the frequency with the highest spectral power.
**ePeriodicity (Li, Wang & Han, 2012)** They proposed the discrepancy score for each candidate period, and the result is the max probability ${p_i}(T)$ which is the proportion of observations concentrating on a set of time points through segmented by the candidate period to all observations observations.
**PRED (Yuan et al., 2017b)** They use a probability generation model of the time gap between two consecutive records to detect the true period. We can get the results by Gibbs sampling.

The parameters of above baseline methods are set to their suggested values. In our proposed Algorithm 1, we set the maximal potential period ${T_{max}}$ to half the length of input sequence *X*.

The accuracy is computed by relating FP (False Positives), FN (False Negatives), TP (True Positives) and TN (True Negatives), and defined as Eq. (21)

(21) $$Accuracy = {{TP + TN} \over {TP + TN + FP + FN}}$$

For each parameter setting of the synthetic time sequence, we repeat the experiment for $1,\!000$ times and report the percentage of correct period detections as the *accuracy*.

#### Effectiveness of relative entropy

In previous works, entropy was employed as a measurement for evaluating periodicity, despite its known bias toward favoring the shorter periods. In this section, we will elucidate this issue and demonstrate the effectiveness of the relative entropy as an alternative measure.

**Figure 6:**
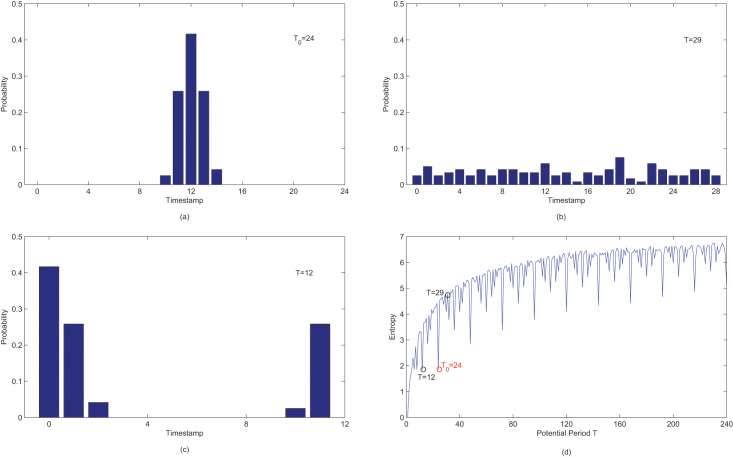
(A–D) Probability distributions and entropy values of observations in *X* after segmenting by potential period *T*.

Based on the work of Li, Wang & Han (2012) work, the binary sequence *X* is more concentrated when segmented by the true period ${T_0}$ compared to an incorrect period ${T_f}$. Consequently, entropy *H* serves as a measure of concentration, with the entropy of the true period ${T_0}$ being lower than any incorrect period ${T_f}$. To illustrate this, We use a data generator, as described in the “Experimental Settings” section, to generate a periodic behavior binary sequence *X* consisting of $2,\!880$ observations with the true period ${T_0} = 24$. In Figs. 6A–6C display the distribution of observations when a binary sequence *X* with the true period ${T_0} = 24$ is segmented by potential periods $24$, $29$ and $12$. Figure 6D illustrates the entropy for different potential periods. Notably, in Fig. 6D, the entropy corresponding to the true period ${T_0} = 24$ is smaller than the incorrect period ${T_f} = 29$, and the corresponding distribution in Fig. 6A is more concentrated than the one in Fig. 6B. Thus, it seems that entropy can be employed as a criteria for periodicity detection, though it could favor shorter periods.

The distribution ${p_i}(T)$ in Fig. 6A is identical to that in Fig. 6C. As demonstrated in Fig. 6D, it is evident that the values of $H(T = 12)$, $H(T = 6)$ and $S(T = 3)$ are all equal to the true period $H({T_0} = 24)$. This could potentially leads to a wrong conclusion that $T = 3$ is the true period. This problem arises due to the lack of a reference for comparing ${p_i}(T)$ among different potential period *T*. The distributions related to different potential periods can not be directly compared.

In fact, if observations fall in a narrow interval after being segmented by a potential period *T*, the distribution is more peaked in contrast to the uniform distribution of *T*. As depicted in Fig. 6, although the entropy of Fig. 6A is equal to entropy of Fig. 6C, the distribution of ${p_i}({T_0} = 24)$ is more peaked than ${p_i}(T = 12)$. Thus, it is feasible to utilize the uniform distribution as a reference and employ the entropy discrepancy between ${p_i}(T)$ and the uniform distribution of a potential period *T* as criteria for periodicity detection.

**Figure 7:**
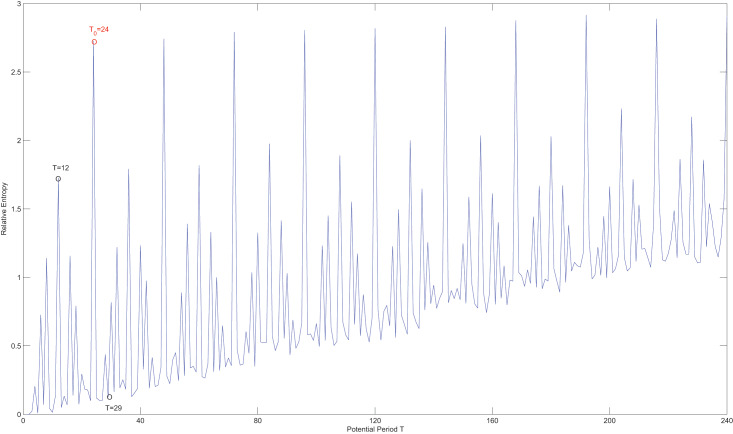
The relative entropy of different candidate periods.

In Fig. 7, it is evident that relative entropy reaches it maximum when the potential period *T* is equal to the true period ${T_0}$. Therefore, the new measurement effectively mitigates the issue of favoring shorter periods. Although the maximum relative entropy can be also observed when $T = k{T_0},k \in {N}$, the true period ${T_0}$ can still be accurately obtained using Algorithm 1.

#### Results and analysis

We study the performance of the compared methods on the synthetic dataset byvarying one parameter in each experiment while keeping the others at their default values.

**Figure 8:**
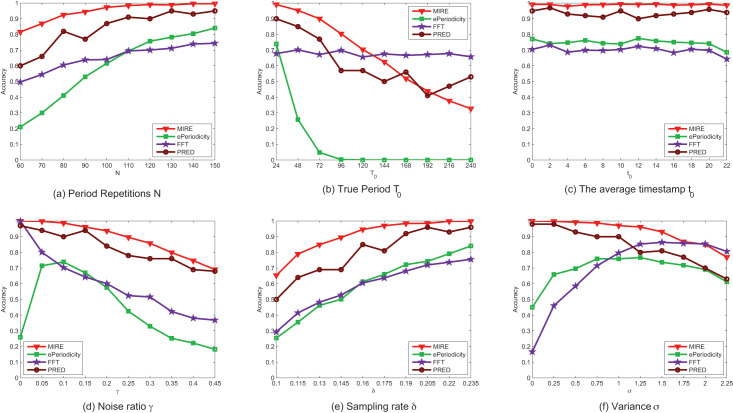
(A–F) The performance comparison on synthetic data with various parameter settings.

Figure 8 displays the performance of the compared methods on synthetic dataset. It illustrates that, in most case, our method exhibits higher accuracy than the comparison methods. Figure 8 also reveals that most methods perform better when the data is of higher quality, characterized by factors such as a larger number of period repetitions *N*, a higher sampling rate $\delta$, lower noise rate $\gamma$ and smaller variance $\delta$.

The accuracy of ePeriodicity is consistently reliable with the default parameters only when the observations contain more than $150$ periods, which might not be hold in real spatiotemporal datasets. In contrast, our method achieves close to $100\%$ accuracy when the observations contains $120$ periods. Impressively, our method can detect more than $80\%$ of the periods even with as few as $60$ observed periods, a scenario common in many spatiotemporal datasets, such as check-in dataset (Cho, Myers & Leskovec, 2011) and Smart Card Data (SCD) of public transport (Itoh et al., 2014).

The performance of all methods, except FFT, deteriorates as ${T_0}$ increases, probably due to increased noise interference in longer periods. Due to bias toward shorter periods, ePeriodicity fails to detect period when the true period ${T_0}$ exceeds $72$.

The center of observed time $t$ has negligible effect on the performance of all methods and can be considered as the default value of parameters. In our experiment scenario, where there is only one mobility intention in a period, the performance of ePeriodicity is no better than FFT, while PRED serves as the best baseline method. However, their method is time-consuming for involving Gibbs sampling.

Figures 8D and 8E demonstrate that as the noise rate $\gamma$ decreases and the sampling rate $\delta$ increases, all methods achieve better results in period detection. Figure 8F illustrates that it becomes considerably more challenging to detect the true period when there is significant oscillation. For instance, when $\delta = 2.25,{T_0} = 24$ and ${t_0} = 12$, approximately $99\%$ of observations fall within the interval $[4,\!18]$, which covers $60\%$ of ${T_0}$. In this extreme case, the distribution generating the time sequence lacks a steep peak, and the performance of MIRE is no better than that of FFT.

In conclusion, these results indicate that the proposed periodicity detection method is better suited for detecting periodicity than the other compared methods.

### Performance evaluation using real dataset

We evaluated the performance of our proposed human periodic behavior mining model using two real spatiotemporal datasets: ThaiLandData and Gowalla. We have followed the tensor construction method outlined in Eq. (2) and employed the CP decomposition algorithm provided by TensorLy (https://tensorly.org/) (Kossaifi et al., 2019) to decompose the tensors obtained from the two datasets.

The ThaiLandData dataset is a social media check-in dataset collected from Foursquare Swarm, a mobile app that enables users to share their locations with their friends. The dataset was collected by a researcher interested in tourism data mining as part of the Team for Universal Learning and Intelligent Processing (TULIP) research gruop. The dataset encompasses records primarily located in Thailand and spans from $2014$ to $2018$. It consists of $423,\!991$ records contributed by $3,\!046$ users. The majority of the dataset is generated by tourists, and the records appear to be random in both time and place, lacking clear periodicity. However, our methods, as presented in this article, revealed some intriguing insights from this seemingly non-periodic data.

From the ThaiLandData dataset, our proposed method extracted ten mobility intentions. For the parameters of the AdaBoost classifier used for mapping check-ins to mobility intentions, we configured the base estimator as an MLP. The maximum number of estimators was set to 100, and the learning rate was assigned a value of 2.0. Additionally, the Boosting algorithm was set to SAMME.R.

We focused on mobility intentions with more than ten observations to investigate their periodicity. Table 1 presents the ratio of users exhibiting periodic mobility intentions among all users.

**Table 1:** Ratio of periods reported by our proposed methods for ThaiLandData.

Accommodation	Affair	Entertainment	Dining	Home
4.2%	20.0%	20.8%	42.8%	4.3%
Recreation	Shopping	Study	Traffic	Visiting
26.9%	44.77%	15.9%	19.5%	28.7%

The “Home” mobility intention, representing the act of returning home after daily activities, is expected to exhibit periodicity. However, only $4.3\%$ of user display periodic patterns, likely due to the dataset primarily comprising tourists. In the context of tourism, “Shopping” and “Dining” are the most common periodic behaviors. Nearly half of the users’ shopping and dining behaviors are periodic, with most of these showing a 24-h periodicity, aligning with the characteristics of tourist activities.

In contrast, the periodicity of “Visiting” is less consistent, ranging from 24 h (1 day) to 168 h (1 week). This suggests that sightseeing is more random compared to shopping and dining. “Accommodation”, although an essential part of tourism, doesn’t exhibit strong periodicity for most users, possibly due to irregular resting patterns during their tours.

**Figure 9:**
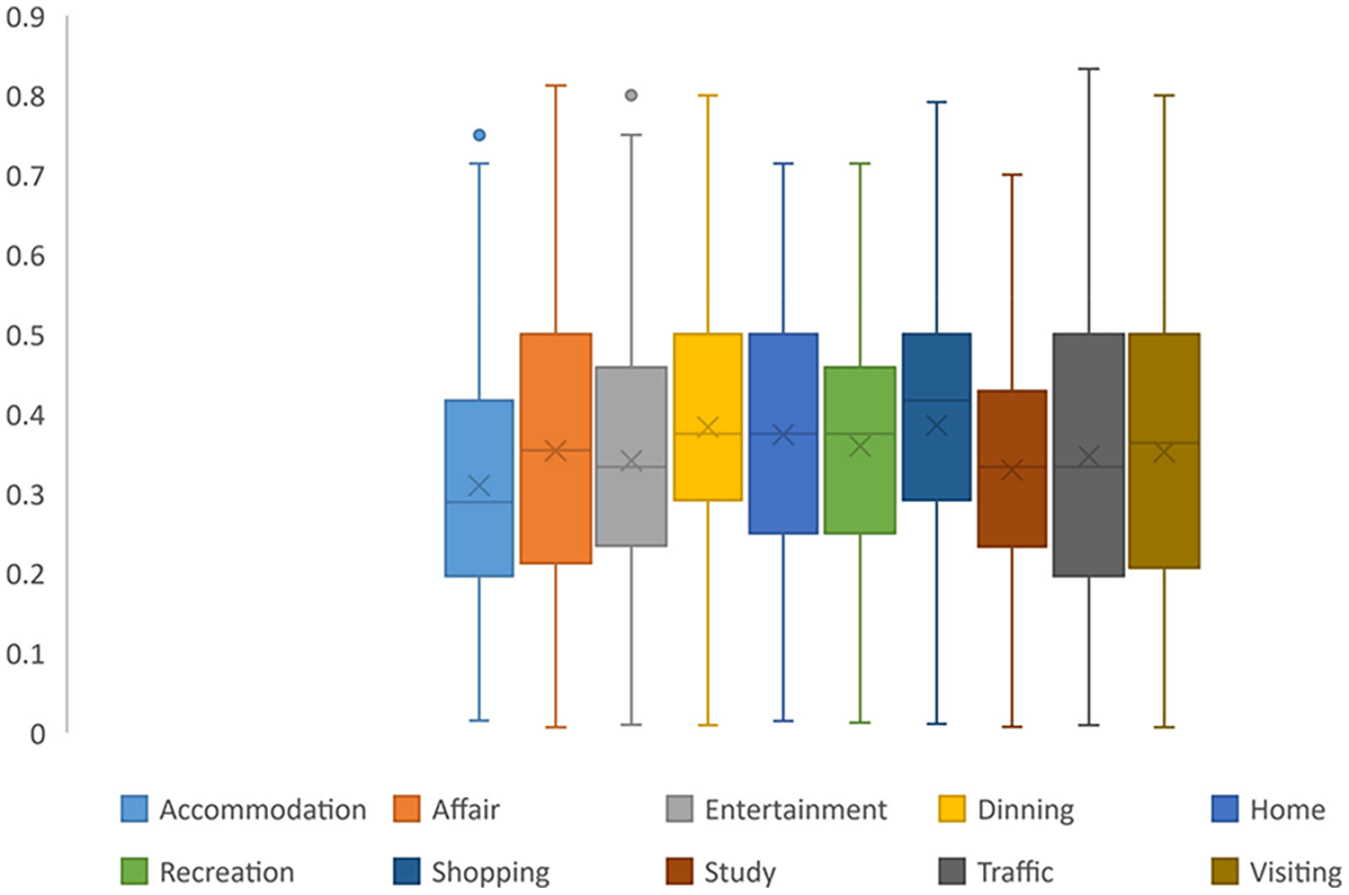
CWC value distribution.

While users’ mobility intentions display periodicity, the degree of periodicity varies, as shown in Fig. 9. As mentioned earlier, “Accommodation” for most users lacks periodicity, but users following a usual routine, which includes “Entertainment” and “study” (such as going to colleges or libraries), exhibit a more distinct periodic pattern. These mobility intentions show a high degree periodicity. In contrast, the periodicity of “Shopping” and “Dining” appears to be less regular.

The Gowalla dataset is a public available social media check-in dataset (Cho, Myers & Leskovec, 2011). It comprises $6,\!442,\!890$ check-ins of $196,\!591$ users from February 2009 to October 2010. After applying tensor decomposition techniques, we successfully extracted $10$ distinct mobility intentions from this extensive dataset. The parameters for the AdaBoost classifier in the Gowalla dataset closely mirror those in the ThaiLandData dataset. However, there are slight variations: the learning rate is adjusted to $0.5$, and the maximum number of estimators is set to $200$.

**Figure 10:**
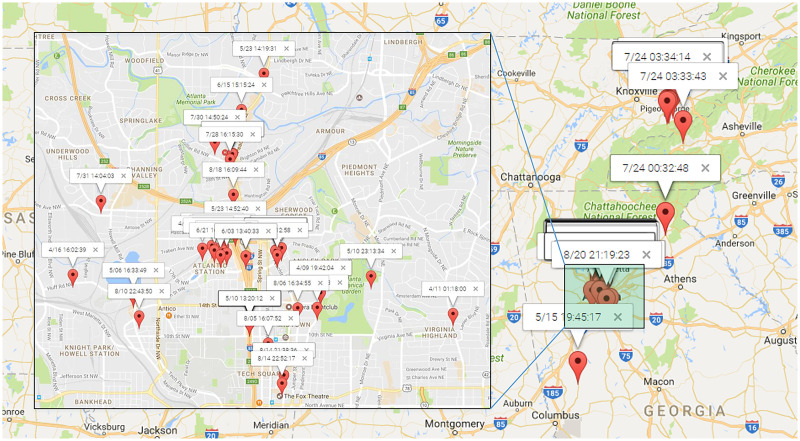
The check-ins of $\# 11838$ which were most in the north of Atlanta.

To illustrate the process of discovering human periodic behavior, We randomly select a user from Gowalla dataset. Figure 10 presents the distribution of $75$ check-ins for this randomly chosen user, identified as user $\# 11838$. These check-ins were recorded from April to August 2010, with the majority of them concentrated in the north area of Atlanta. It is noteworthy that only four locations have been checked twice, making it challenging, if not impossible, to identify direct periodic behavior based on location data alone.

By adopting the trained Adaboost model, we were able to identify a total of seven distinct mobility intentions for the recorded check-ins, as outlined in Table 2. Subsequently, we employed our proposed periodicity mining algorithm to mine human periodic behavior, utilizing the default sampling rate of one hour. The results of this analysis are displayed in Table 2.

**Table 2:** Periods reported by our proposed methods for various mobility intentions in Gawalla.

Mobility intention	Commuting	Daily routine	Dining	Entrainment	Recreation	Shopping	Tour
Observations	25	7	15	2	2	11	13
Periodicity	24	53	24	NA	NA	24	24

Table 2 reveals that most mobility intentions exhibit a periodicity of $24$ h. The majority of observations are associated with the “Commuting” mobility intention, which aligns with the common daily routine and occurs at a $24$-h interval. The “Daily Routine” periodicity is approximately 53 h, indicative of some routine behaviors, like refueling the car, happening every two days. However, we were unable to detect the periodicity of “Entertainment and Recreation” due to an insufficient number of observations.

**Figure 11:**
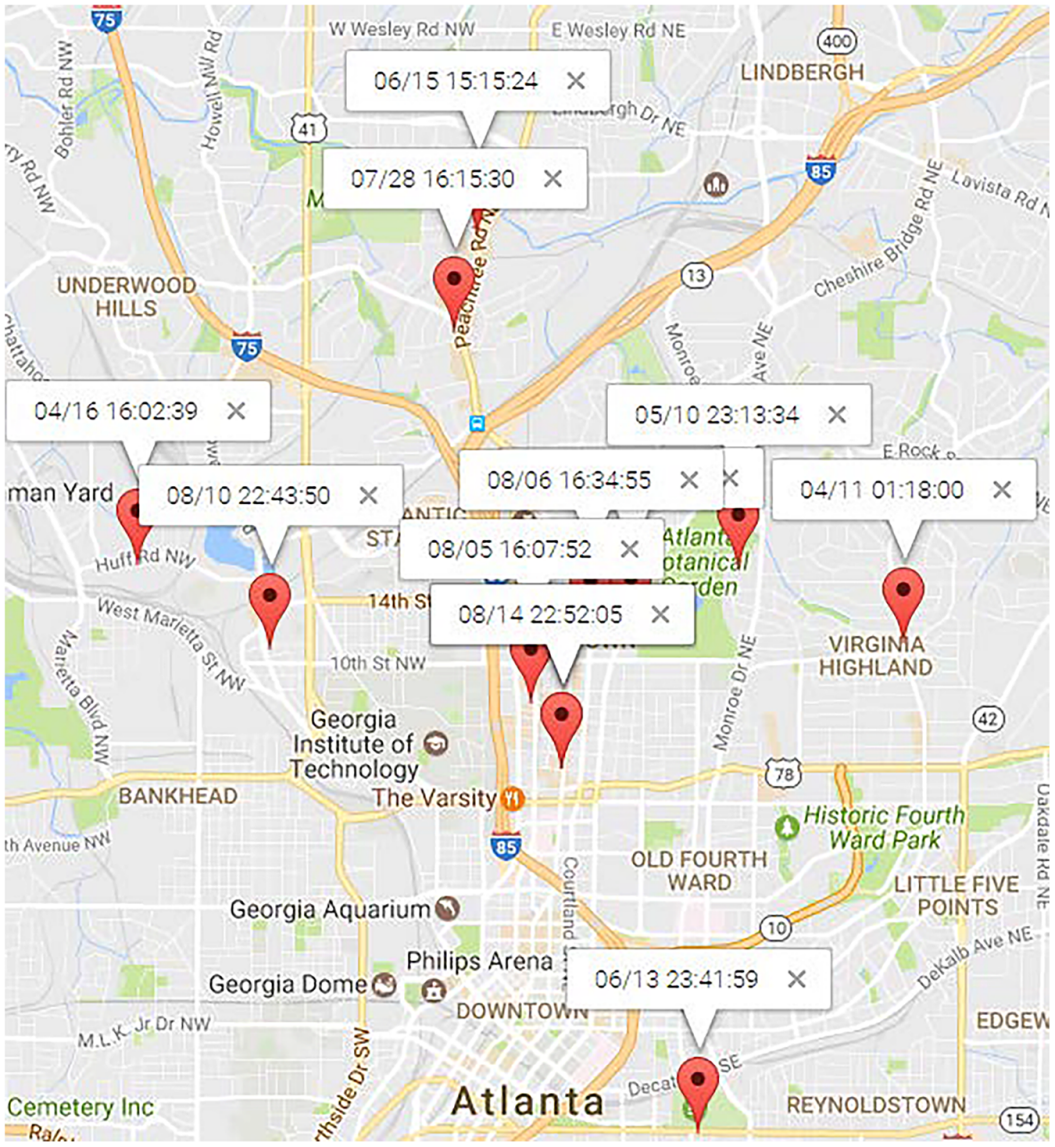
Locations of “dining” topic.

**Figure 12:**
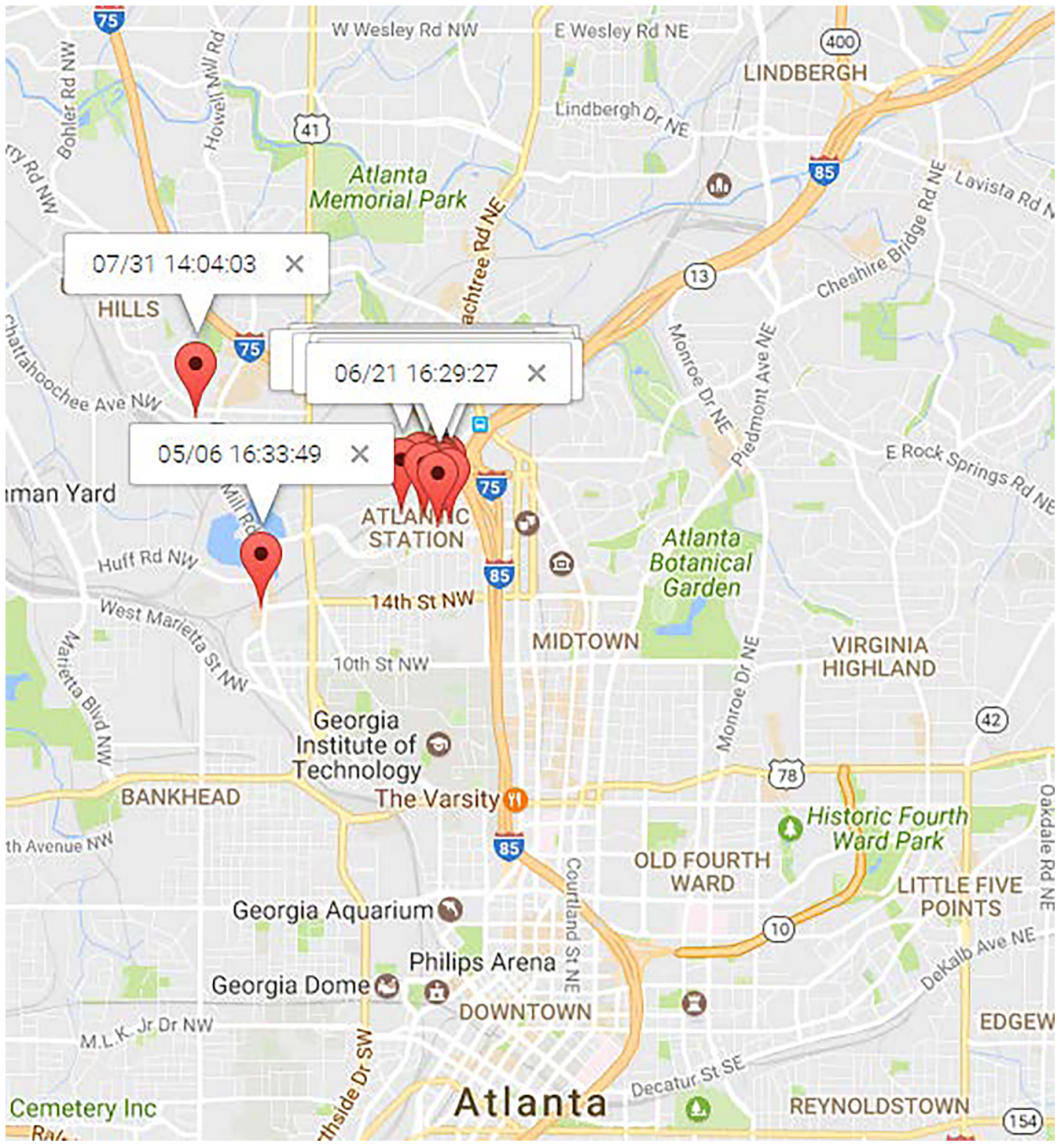
Locations of “shopping” topic.

Figures 11 and 12 illustrate the locations of “Dining” and “Shopping” mobility intention. “Dining” locations are spread across the north area of Atlanta City, as depicted in 11. Traditional mining methods solely based on locations struggle to mine this type of human periodic behavior. For the user $\# 11838$, most of his “shopping” mobility intention occurred at a shopping center, alghough occasional visits to new places for shopping were also observed. PRED (Yuan et al., 2017b) can identify such human periodic behavior through location clustering, but their results may lack precision since they didn’t consider observations from the northwest and southwest in Fig. 12, which is inconsistent with reality. On the contrary, MIRE offers more accurate and insightful results.

Regarding the “Tour” mobility intention, its $24$-h period is somewhat unreliable. This is due to the numerous check-ins within one “Tour” period, which contradicts the assumption that only one mobility intention should occur in a period. We plan to address this issue in our future work.

### Location prediction on real datasets

As aforementioned, mining human period behavior has numerous applications. In this section, we leverage the human period behavior mined from spatiotemporal datasets to predict users’ next locations.

In this context, location prediction refers to forecasting a user’s location at a given time. In the experiments in this subsection, the specified time corresponds to the next timestamp at which a periodic mobility intention is predicted to occur. For our proposed MIRE algorithm, the next location for a user’s periodic mobility intention is the one with the highest historical probability within that periodic mobility intention.

#### Experimental settings

**Datasets and Evaluation Metric:** We employed two real spatiotemporal datasets for location prediction. One is Gowalla check-ins dataset and the other is Beijing Bus Smart Card (BBSC) dataset. The BBSC dataset collects prepaid smart card records for public transportation in Beijing, China. It contains $275,\!951,\!094$ bus transaction records involving $16,\!161,\!460$ users in October of 2014, which contains more than $90\%$ of Beijing urban public traffic lines. To conduct our experiments, we categorized users into five groups based on the sparsity of their records. From both the Gowalla and BBSC datasets, we randomly selected $500$ users from each groups. For training and testing, we allocated $90\%$ of each selected user’s records in chronological order for training, reserving the remaining $10\%$ for testing.

Effectiveness is measured by calculating the averaged error in distance, represented as the Euclidean distance between the actual and the predicted location of a testing record. The final result is the average value derived from all test records.

**Methods for comparison:**

In our experiments, we compared our method with three baseline models: PMM (Periodic Mobility Model) (Cho, Myers & Leskovec, 2011), Periodica (Li et al., 2010) and PRED (Yuan et al., 2017b).

The PMM adopts a Gaussian mixture model which centers at “home” and “work” to model user locations. It employs an independent truncated Gaussian distribution for the temporal component.

The Periodica model employs kernel density estimation (KDE) to extract regions and then estimates the periodicity for each region using a combination of FFT and autocorrelation.

The PRED is discussed in “Periodicity Detection on Synthetic Time Series Data” subsection.

The parameters for the baseline methods were configured according to their recommended settings.

### Performance study

**Figure 13:**
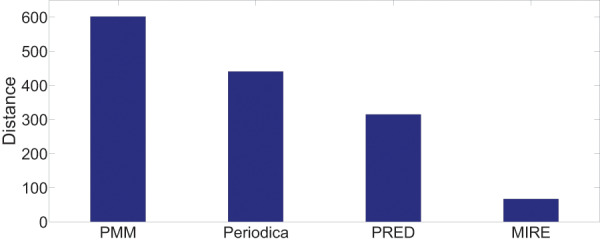
Model performance comparison on the Gowalla dataset.

**Figure 14:**
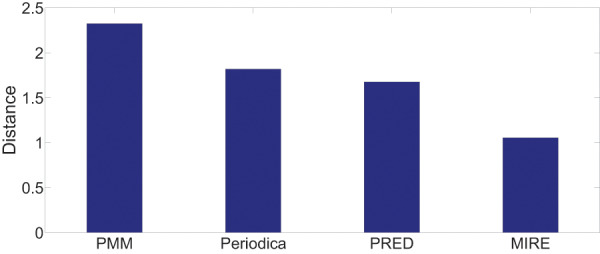
Model performance comparison on the BBSC dataset.

Figures 13 and 14 display the performance on all testing records. In general, for the two datasets, MIRE consistently exhibits significantly lower error distances than the baseline methods. PMM, on the other hand, consistently generates the maximum error.

On the Gowalla dataset in Fig. 13, there is notable performance gap between PMM and the other comparison methods. One possible reason for this is that users frequently visit locations beyond just “work” and “home”, which PMM focuses on. However, this gap is not observed in Fig. 14. The reason for this difference is that in the BBSC dataset, around $70\%$ of users use public transportation to commute between home and work place, aligning well with the assumption of PMM.

Periodica performs better than PMM in Fig. 14. This improvement could be attributed to Periodica’s use of multiple reference spots in addition to “work” and “home”, which aligns more closely with real-world scenarios. PRED, which enhanced the clustering algorithm of Periodica using the Chinese Restaurant Process (CRP) and incorporated a time model as a constrain for clustering, demonstrates the best performance among all baseline models. The proposed MIRE model outperforms the baseline models significantly.

Overall, Figs. 13 and 14 provides a comprehensive and clear comparison of the proposed MIRE model with various baseline methods.

We are also intrigued by the relationship between record density and the performance of human period behavior models. For each dataset, we classify users into five groups based on their record density, which refers to the record count per user per day in the datasets.

**Figure 15:**
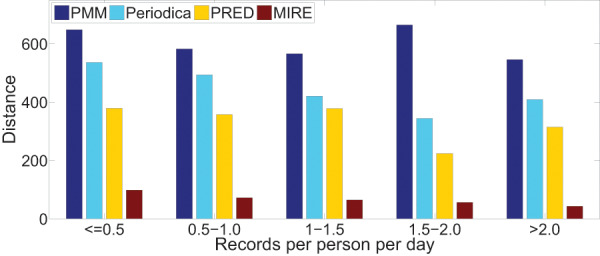
Comparing model performance on the Gowalla dataset with varying record densities.

**Figure 16:**
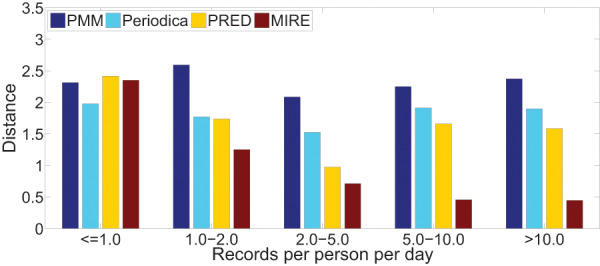
Comparing model performance on the BBSC dataset with varying record densities.

Figures 15 and 16 illustrate the error distance of different models in various data groups. As record density increases, the performance of Periodica and PRED, which are based on location cluster, initially improves and then declines. This pattern might be attributed to models obtaining more information at the start and performing better when human period behaviors are simpler. However, as more records accumulate, resulting in a mixture of more complex human period behaviors, Periodica and PRED struggle to handle this situation. In contrast, MIRE consistently achieves better performance with increasing record density.

## Conclusions

In this article, we proposed a novel human periodic behaviors mining model, MIRE, which incorporates the mobility intention as hidden variable and utilizes relative entropy to detect periodicity. More specifically, our model employs tensor decomposition to uncover mobility intentions from spatiotemporal datasets, allowing us to capture human periodic behavior as generalized spatiotemporal features across diverse users. We have also developed an innovative detection algorithm based on relative entropy and provided a rigorous proof that the maximum relative entropy corresponds to the correct periodicity. Our extensive experiments demonstrate the robustness and superior performance of MIRE compared to existing periodicity detection methods.

The main contributions of this article are:

- We introduce the MIRE model for periodic behaviors mining, which involves the extraction of mobility intentions from spatiotemporal datasets and the training of supervised algorithm to transform historical observations into mobility intention sequences.
- We propose a novel periodicity detection algorithm based on relative entropy, which effectively uncovers periodic patterns.
- We introduce a criterion based on CWC to measure the periodicity quality.

The proposed MIRE model can be applied to various online and offline applications, including recommendation systems, location prediction, public transport schedule optimization, and more. With the knowledge about users’ periodic behavior, such as their shopping habits for commodities like food and cooking oil, businesses can make more precise recommendations and tailor advertising to users’ preferences. Many potential applications stand to benefit from the high accuracy of the MIRE model.

There are several interesting directions for future researchf. For instance, in the “Period Identification” section, we assume that mobility intention sequences are periodic. However, not all mobility intention sequences exhibit periodicity. It’s possible to use the relative entropy to determine whether a mobility intention sequence is periodic. In this article, we assume that there is only one mobility intention in a period, and MIRE is designed for this assumption. An important extension would be to handle cases with multiple observations in each period using relative entropy.

## Supplemental Information

10.7717/peerj-cs.1851/supp-1 Supplemental Information 1 The source code for periodicity detection on synthetic time series data and performance evaluation using real dataset. DataGen.m is for producing synthetic time series dataset and other for intention detection such as shopping detection.

## Additional Information and Declarations

### Data Availability

The following information was supplied regarding data availability: The Gowalla dataset is available at SNAP: https://snap.stanford.edu/data/loc-gowalla.html.
